# Peripheral Parenteral Nutrition Solutions and Bed Bath Towels as Risk Factors for Nosocomial Peripheral Venous Catheter-related Bloodstream Infection by *Bacillus cereus*

**DOI:** 10.7150/ijms.82054

**Published:** 2023-03-05

**Authors:** Chieko Hino, Masakazu Ozaki, Takashi Kitahara, Kyoji Kouda, Kyoko Shikichi, Itaru Nakamura, Shinya Kawai, Shigeharu Oie

**Affiliations:** 1 Clinical Pharmacology, Graduate School of Medicine, Yamaguchi University, Japan.; 2 Pharmacy Department, Yamaguchi University Hospital, Japan; 3 Department of Pharmacy, Hofu Institute of Gastroenterology, Japan; 4 Clinical Laboratory, Yamaguchi University Hospital, Japan; 5 Department of Infection Prevention and Control, Tokyo Medical University Hospital, Japan; 6 Faculty of Pharmaceutical Sciences, Sanyo-Onoda City University, Japan

**Keywords:** venous catheter -related bloodstream infection, *Bacillus cereus*, peripheral parenteral nutrition, microbial growth

## Abstract

In Japan, China, and Singapore, several studies have reported increased incidences of peripheral venous catheter-related bloodstream infection by *Bacillus cereus* during the summer. Therefore, we hypothesized that bed bathing with a *B. cereus*-contaminated “clean” towels increases *B. cereus* contact with the catheter and increases the odds of contaminating the peripheral parenteral nutrition (PPN). We found that 1) professionally laundered “clean” towels used in hospitals have *B. cereus* (3.3×10^4^ colony forming units (CFUs) / 25cm^2^), 2) *B. cereus* is transferable onto the forearms of volunteers by wiping with the towels (n=9), and 3) *B. cereus* remain detectable (80∼660 CFUs /50cm^2^) on the forearms of volunteers even with subsequent efforts of disinfection using alcohol wipes. We further confirmed that *B. cereus* grow robustly (10^2^ CFUs /mL to more than 10^6^ CFUs /mL) within 24hours at 30°C in PPN. Altogether we find that bed bathing with a towel contaminated with *B. cereus* leads to spore attachments to the skin, and that *B. cereus* can proliferate at an accelerated rate at 30°C compared to 20°C in PPN. We therefore highly recommend ensuring the use of sterile bed bath towels prior to PPN administration with catheter in patients requiring bed bathing.

## Introduction

*Bacillus cereus* is a type of spore-producing bacteria that is often detected in air, soil, dust, water, and food 1. Furthermore, this type of bacteria may cause relatively mild food poisoning. In addition, several studies reported that the use of bed bath towels or sheets contaminated with *B. cereus* induced peripheral venous catheter-related bloodstream infection (PVC-BSI) 2-5. On the other hand, when *B. cereus* is detected in sterile samples of the human body, the spores are extensively distributed in a hospital environment; therefore, the situation is often regarded as contamination. However, an increasing number of studies suggested that *B. cereus* causes serious septicemia (bacteremia) 6-21. In addition, the administration of peripheral parenteral nutrition containing glucose, amino acids, and electrolytes (PPN) 22-25 and the summer season 26-28 have been indicated as risk factors for *B. cereus*-related septicemia. Of these risk factors, the former was extracted because *B. cereus* may proliferate in PPN. However, the reason why *B. cereus*-related septicemia frequently develops in the latter, the summer season, remains to be clarified. In this study, we examined the etiology of a high incidence of *B. cereus*-induced PVC-BSI in the summer.

## Materials and Methods

### Microorganisms employed

The following strains were used: *B. cereus* ATCC 11778, a total of 28 clinical isolates of *B. cereus* from blood (isolated in A university hospital and B university hospital), *E. coli* ATCC 25922, *Klebsiella pneumoniae* IFO 3318, *Pseudomonas aeruginosa* ATCC 27853, *Serratia marcescens* IFO 3046, and *Candida albicans* IFO 1386.

### Test solutions

3 types of PPN ((Bfluid^®^ Injection, Otsuka Pharmaceutical Factory, Inc., Tokushima, Japan; Paresafe^®^ and Pareplus^®^, AY Pharm, Co., Tokyo, Japan), soybean oil (Intralipos^®^ 20%, Otsuka Pharmaceutical Factory, Inc.), albumin (Albumin 25% i.v. 5 g/20 mL- Benesis^®^, Japan Blood Products Organization., Tokyo, Japan), acetated Ringer's solution (Solacet^®^ F, Terumo Co., Tokyo, Japan), normal saline (Isotonic Sodium Chloride Solution “Hikari^®^”, Hikari Pharmaceutical Co., Tokyo, Japan), 5% glucose (5% Glucose Injection PL "Fuso^®^", Fuso Pharmaceutical Industries, Ltd. Osaka, Japan), distilled water (Sterile Water for Injection^®^, Hikari Pharm. Co.), and 2 kinds of total parenteral nutrition (Elneopa-NF^®^ No.2 Injection, Otsuka Pharmaceutical Factory, Inc., ; Fulcaliq^®^ 2. Terumo Co.,) were used.

### Viability of microorganisms in infusion solutions

All microbes used in the experiment were cultured on trypticase soy agar (TSA; Eiken Chemical Co., Tochigi, Japan) for 1-2 days at 35ºC, scraped into sterile phosphate-buffered saline (PBS), and centrifuged three times at 3,000 rpm for 10 min to remove the growth medium. Resuspension was carried out in PBS, yielding a concentration of approximately 10^4^-10^5^ colony forming units (CFUs)/mL. Next, 0.05 mL of the resuspension was added to 4.95 mL of each infusion solution. The test solutions were incubated at 20 and 30ºC, and plate counts were performed at 6, 24, 48 h. Each sample was diluted 10-, 10^2^-, 10^3^-, 10^4^-, 10^5^-, and 10^6^- fold in normal saline. Pipettes were used to transfer 0.25 mL of undiluted or diluted samples to TSA. The plates were streaked with a glass 'hockey stick' and incubated at 35ºC for 1-2 days for measurement of the number of viable microorganisms. Each experiment was repeated 3 times and the mean of the 3 repeats was calculated.

### Contaminated towels with *B. cereus*

We had initially observed that hospital bed bath towels shipped from the laundry service factory yield *B. cereus*. The bed bath towels were prepared by the laundry service, washing in high temperature at 80 ºC for 10 minutes, per regulatory guidelines in Japan. Approximately 1.3 × 10^3^ CFUs / cm^2^ of *B. cereus* was found on the towels.

### Transfer of *B. cereus* from a contaminated towel to the skin, and disinfection of *B. cereus* on the skin by alcohol

This study was conducted with the participation of volunteer pharmaceutical students and staff from the Sanyo-Onoda City University Ethics Review Committee (Title: Attachment of *B. cereus* to the skin related to bed bathing with *B. cereus*-contaminated towels, Approval date: September, 2022, Examination certificate management number: 22001).

In 12 subjects (volunteers), we initially screened for presence or absence of *B. cereus* on subject skin by wiping bilateral forearms (5 cm × 10 cm) with a piece of wet sterile gauze (5 cm × 5 cm). This gauze was placed in a bottle containing 20 mL of sterile physiological saline, and ultrasonically treated at 37kHz for 5 min. Subsequently, the solution in the bottles was diluted 10- fold in normal saline. Pipettes were used to transfer 1 mL (0.25 mL × 4) of undiluted or diluted samples to PBCW agar (Eiken Chemical Co., Ltd.) containing egg yolk. The plates were streaked with a glass 'hockey stick' and incubated at 35ºC for 24 h. Additionally, the residual solution was passed through 0.22 µm membrane filters, 5 cm in diameter (Thermo Scientific Nalgene Analytical Filter Unit 0.2 µm, Thermo Scientific, Wilmington, NC, USA), and the filters were placed on PBCW agar containing egg yolk. After incubation at 35ºC for 24 h, the colonies of *B. cereus* were counted 29.

Next, the following experiment was conducted in the 9 volunteers that we found *B. cereus* to be absent on the forearms. Two pieces (5 cm × 5 cm) of bed bath towel that were found to be contaminated with 1.3 × 10^3^ CFUs/cm^2^ of *B. cereus* were each dampened with 2mL of sterile purified water. Using each piece, each subject's bilateral forearms (5 cm × 10 cm) were wiped. After naturally air drying, the unilateral forearm was wiped with sterile-water-drenched gauze, and the amount of *B. cereus* in this gauze was investigated to calculate the forearm *B. cereus* 30. Additionally, the other forearm was wiped twice with a medical-grade absorbent cotton (4 cm × 8 cm) containing 1.6 mL of ethanol for disinfection (76.9-81.4 vol%), and wiped with a piece of sterile-water-drenched gauze after 1 min. Subsequently, the amount of *B. cereus* in this gauze was investigated to calculate the forearm CFUs of *B. cereus* after alcohol disinfection. The amount of *B. cereus* in the gauze used for wiping was calculated as described above. In 9 volunteers, this test was conducted twice. The forearm CFUs of *B. cereus* before and after alcohol disinfection was tested using Wilcoxon's signed-rank test.

## Results

**Figure 1:**
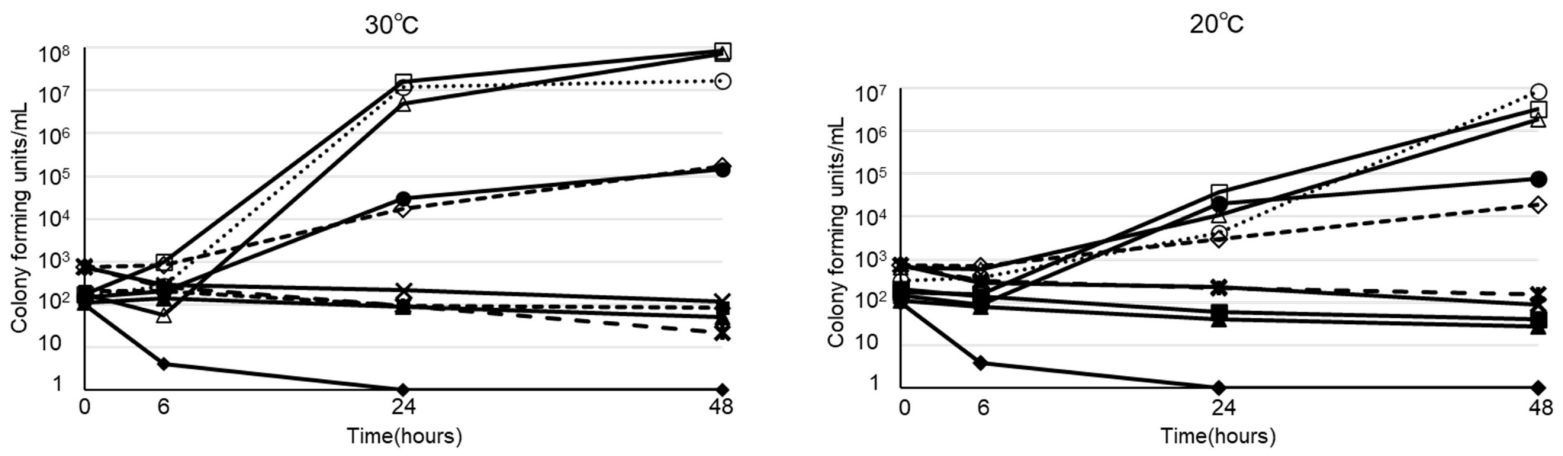
Viability of *Bacillus cereus* in peripheral parenteral nutrition (Bfluid^®^ (○), Paresafe^®^ (△), Paleplus^®^ (□)), 5% albumin (◇), soybean oil (●), normal saline (▲), acetated Ringer's solution (■), 5% glucose (◆), and total parenteral nutrition (Elneopa-NF^®^ No. 2 (⨯), Fulcaliq^®^ 2 (⁎))

**Figure 2:**
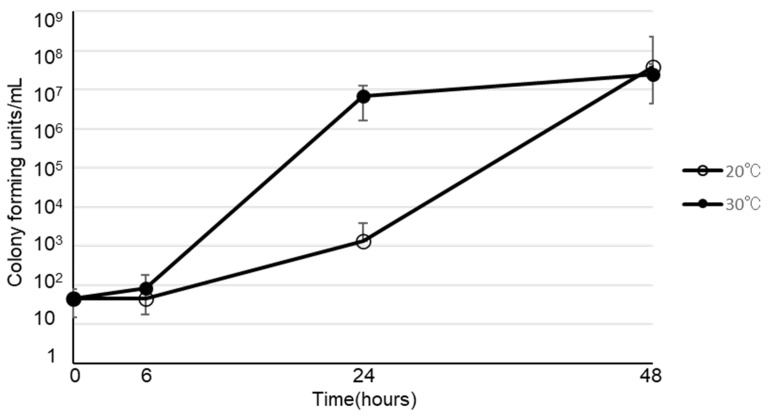
Viability of twenty-eight strains of clinically isolated *Bacillus cereus* in peripheral parenteral nutrition (Bfluid^®^)

The viability of *B. cereus* in various types of infusion fluid are shown in Figure 1. At 30ºC, *B. cereus* promptly proliferated in all 3 types of PPN we tested. Furthermore, *B. cereus* also proliferated in soybean oil or albumin, but not in acetated Ringer's solution, normal saline, 5% glucose, or total parenteral nutrition. On the other hand, the growth rate at 20ºC was slower than at 30ºC. However, *B. cereus* proliferated robustly by the 48-hour timepoint in PPN, soybean oil, and albumin, as demonstrated at 30ºC. The viability of 28 clinical isolates of *B. cereus* in PPN (Bfluid^®^) are shown in Figure 2. These isolates promptly proliferated in PPN, as shown for a standard strain of *B. cereus* in Figure 1. After 24 h, these isolates had more rapidly proliferated at 30ºC, but the CFU of bacteria after 48 h was similar between 30ºC and 20ºC.

**Figure 3:**
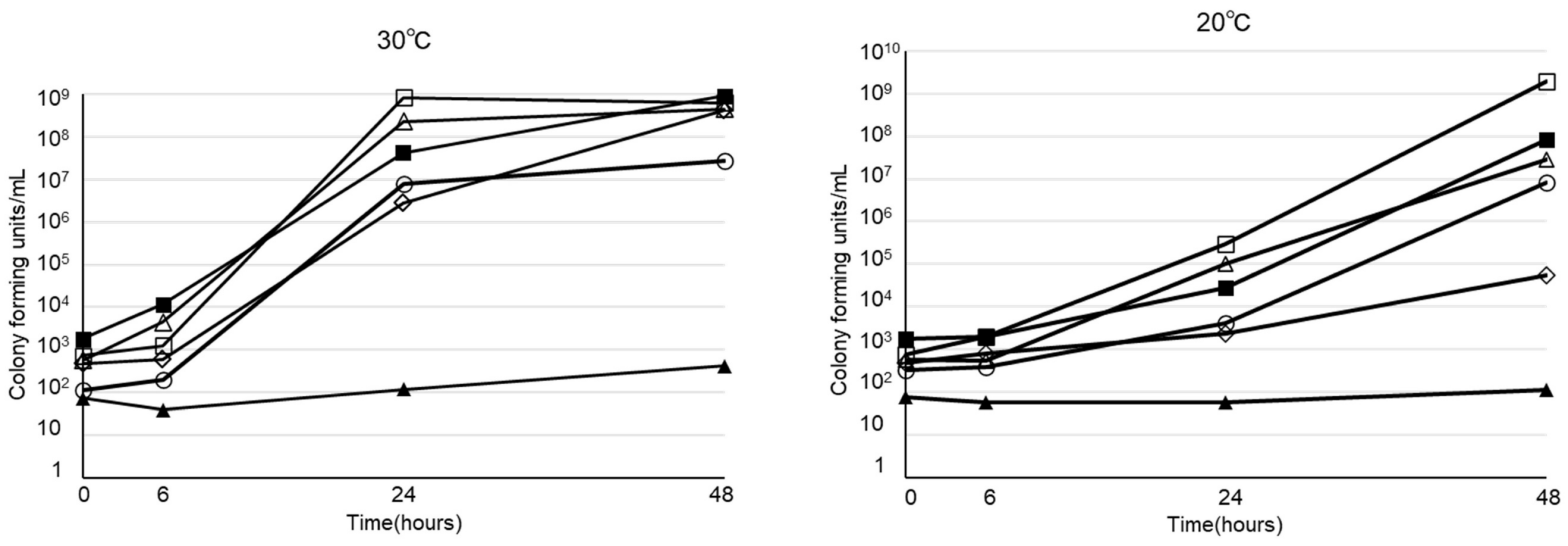
Viability of *Bacillus cereus* (○)*, E. coli* (△),* Klebsiella pneumoniae* (□),* Pseudomonas aeruginosa* (◇),* Serratia marcescens* (■), *Candida albicans* (▲) in peripheral parenteral nutrition (Bfluid^®^) at 30°C and 20°C

The viability of various microorganisms in PPN (Bfluid^®^) at 30ºC and 20ºC are shown in Figure 3. Of the 5 strains that we investigated, 4 (*E. coli*, *K. pneumoniae*, *P. aeruginosa, S. marcescens*), excluding* C. albicans*, promptly proliferated in PPN at 30ºC and 20ºC, as demonstrated for *B. cereus*.

In 3 of the 12 volunteers, 5, 9, and 17 CFUs of *B. cereus* were detected from the right forearm (50 cm^2^) and left forearm (50 cm^2^), respectively. However,* B. cereus* was not detected in any of the other 9 subjects. An experiment was conducted in these 9 volunteers. The amount of *B. cereus* transferred to the skin from the bed bath towel contamination with *B. cereus* was confirmed in 9 subjects shown in Table 1 (upper row: the right hand was wiped with alcohol, lower row: the left hand was wiped with alcohol). After forearm (50 cm^2^) wiping with a towel contaminated with 3.3×10^4^ CFUs /25 cm^2^ of *B. cereus*, 240 to 1,260 (median 540) CFUs /50 cm^2^ of *B. cereus* was detected on the left forearm, and 260 to 3,200 (median 760) CFUs /50 cm^2^ of *B. cereus* on the right forearm. Even after disinfection with alcohol, 80 to 620 (median 240) CFUs /50 cm^2^ of *B. cereus* was attached to the right forearm, and 120 to 660 (median 320) CFUs /50 cm^2^ of *B. cereus* to the left forearm. Disinfection with alcohol significantly decreased the amount of *B. cereus* found on the skin (*p* < 0.05, Wilcoxon's signed-rank test). The median values for* B. cereus* even after ethanol disinfection as CFUs per cm^2^ on the left arm and right arm was calculated as 6.4 CFUs /cm^2^ and 4.8 CFUs /cm^2^ respectively.

## Discussion

In Japan, double-bag-type PPN products are routinely used. It was shown that a standard strain of *B. cereus* and its clinical isolates rapidly proliferated in 3 double-bag-type PPN products commercially available in Japan. The growth rate with PPN was equally robust as with fat emulsion or blood preparations (albumin). In this study we demonstrated that* B. cereus* proliferates in PPN, in addition to other organisms such as *E. coli*, *S. marcescens*, *K. pneumoniae*, and *P. aeruginosa,* reported by Omotani et al. 31. In the CDC Guideline for the Prevention of Intravascular Catheter-Related Infections, it is prescribed that tubing used to administer blood, blood products, or lipid emulsion should be replaced within 24 h after the initiation of infusion 32. Such a regulation may be necessary for PPN products (excluding one-bag-type acidic-pH products).

We had initially observed that hospital bed bath towels shipped from the laundry service factory yield *B. cereus*. The bed bath towels were prepared by washing in high temperature at 80ºC for 10 min, per regulatory guidelines in Japan. We determined that the washed “clean” bed bath towels we tested harbored approximately 1.3×10^3^ CFUs/cm^2^ of *B. cereus*, despite undergoing recommended cleaning protocol set by industry-wide practice. Using these “clean” towels to wipe the volunteer forearms, we determined that 6.4 CFUs (median)/cm^2^ of *B. cereus* spores remained on the left and 4.8 CFUs (median)/cm^2^ on the right skin after wiping with contaminated bed bath towels, despite disinfecting the skins with ethanol wipes afterwards. Our calculation of *B. cereus* contamination via catheter placement, using the median CFUs /cm^2^ values, is the following: If a venous indwelling needle (22 G; catheter's cross-sectional area, 0.64 mm^2^) is inserted to the skin, since the cross-sectional area of the catheter is 0.64 mm^2^, *B. cereus* spores may contaminate the indwelling needle at a probability of 0.41% on the left arm and 0.31% on the right arm. If *B. cereus* contaminates the needle or the site of catheter placement, bloodstream infection is a possible outcome due to rapid proliferation in PPN.

The amount of *B. cereus* found in the bed bath towel used in this experiment was approximately 10^3^ CFUs /cm^2^. However, in Japan with a climate of high temperature and high humidity, it is not rare that towels after washing in a cleaning factory may still harbor a higher concentration (10^4^ CFUs /cm^2^) of *B. cereus*. If a towel contaminated with approximately 10^4^ CFUs /cm^2^ of *B. cereus* was used for a bed bath, the probability at which *B. cereus* may access the venous indwelling needle will potentially be higher at 3.1 - 4.1% even after wiping the skin with ethanol. Ethanol is ineffective for killing* B. cereus* spores, and we show here that *B. cereus* has the potential to proliferate in the infusion fluids.

The detection rate of *B. cereus* in blood culture-positive samples is 1.2% according to the information published from the Japan Nosocomial Infections Surveillance (JANIS) in 2020. The rates in A and B Hospitals (736 and 904 beds, respectively) were 1.7 and 22.2%, respectively 28. It is highly probable that bed bath towels contaminated with *B. cereus* may have contributed to the high outbreak rates of *B. cereus* in B Hospitals measured in the blood-culture-positive samples. Patients positive for *B. cereus* on blood culture were mostly patients requiring bed baths. B Hospital's bed bath towels used for bed bathing were from a cleaning factory. Several studies indicated that bed bath towel contamination with *B. cereus* was frequent in the summer of Japan, China, and Singapore 3-5, 33. This may be because towels are often contaminated with *B. cereus* in the summer of Japan, China, and Singapore with a climate of high temperature and high humidity. The bed bath towels used in A Hospitals were independently confirmed by us to have absence of contamination with *B. cereus*.

*B. cereus* is regarded as lowly-pathogenic environmental bacteria 34. However, if infusion fluids such as PPN, is contaminated with *B. cereus*, this type of bacteria may proliferate, and large numbers of bacteria may invade the body, leading to a serious outcome. We encountered a patient with sepsis in whom 1.0×10^7^ CFUs/mL of *B. cereus* was detected in the route of administration during PPN administration 17. Furthermore, several studies indicated that *B. cereus* sepsis was very serious 6, 7, 12, 14, 15, 16, 19, 20, 35, that there was no treatment response despite the administration of effective antimicrobial drugs 9, 21, and that catheter removal was required 8, 36, suggesting intra-route infusion fluid contamination with bacteria. In many hospitals, infusion fluid contamination with microorganisms during administration is not investigated and is often overlooked. However, physicians must recognize that infusion fluid may be contaminated with microorganisms during use.

We previously reported that measurement of the adenosine triphosphate (ATP) level was useful as a simple method to evaluate infusion fluid contamination with microorganisms 17, 37. Since infusion fluid contamination can be estimated in a few minutes by measuring the ATP level, infusion fluid that is being administered should be checked using a simple detection method with ATP in patients in whom sepsis is suspected based on findings, such as a high procalcitonin level.

The inadvertent use of contaminated “clean” towels must be avoided, and to ensure quality control, laundry services for hospitals should be required to pass periodic pathogen screening to be given permission to supply and stock sterile towels. In addition, disposable wipes in turn may be particularly useful in the summer seasons. The implementation of standard guidelines that indicate use of sterile bed bath towels or disposable wipes for patients that require PPN would be beneficial for avoiding preventable infections.

## Conclusion

The use of *B. cereus*-contaminated bed bath towels and administration of double-bag-type PPN products may be involved in higher incidence of *B. cereus* sepsis especially during the summer months in countries such as Japan, China, and Singapore. We demonstrate the ability of *B. cereus* to proliferate rapidly in PPN products and that higher temperatures lead to faster proliferation rate. If double-bag-type PPN products are contaminated with *B. cereus*, it is possible that *B. cereus* may proliferate rapidly in the host via catheter access point. Therefore, the use of sterile bed bath towels must be strongly considered for patients that require PPN.

## Figures and Tables

**Table 1:** Colony forming units of *Bacillus cereus* from the skin of the forearms (50 cm^2^) of 9 subjects after application of bed bathing with *B. cereus*-contaminated towels (3.3×10^4^ cfu/25 cm^2^)

Subject No.	Before wiping with alcohol	After wiping with alcohol
1	320	480
	1000	120
2	240	240
	400	320
3	240	80
	3200	320
4	560	160
	1400	240
5	540	260
	620	540
6	480	120
	760	180
7	640	120
	260	420
8	720	240
	760	200
9	1260	620
	1200	660
Median*	540	240*^1^
	760	320*^2^

*^1^ *p* =0.02071, *^2^ *p* =0.02077 (Wilcoxon signed-rank test)
